# Mutations in the G-domain of Ski7 cause specific dysfunction in non-stop decay

**DOI:** 10.1038/srep29295

**Published:** 2016-07-06

**Authors:** Wataru Horikawa, Kei Endo, Miki Wada, Koichi Ito

**Affiliations:** 1 Department of Computational Biology and Medical Sciences, Graduate School of Frontier Sciences, The University of Tokyo, Kashiwa-city, Chiba 277-8562, Japan; 2 Institute for Virus Research, Kyoto University, Kyoto 606-8507, Japan; 3 Technical office, The Institute of Medical Science, The University of Tokyo, Minato-ku, Tokyo 108-8639, Japan

## Abstract

Ski7 functions as a cofactor in both normal mRNA turnover and non-stop mRNA decay (NSD) mRNA surveillance in budding yeast. The N-terminal region of Ski7 (Ski7_N_) interacts with the ski-complex and the exosome. The C-terminal region of Ski7 (Ski7_C_) binds guanine nucleotides and shares overall sequence and structural homology with the proteins of the translational GTPase superfamily, especially the tRNA/tRNA-mimic carrier protein subfamilies such as EF1α, eRF3, and Hbs1. Previous reports showed that Ski7_N_ polypeptide functions adequately *in vivo*, while Ski7_C_, if any, only slightly. Furthermore, Ski7_C_ does not exhibit GTP-hydrolysing activities under normal conditions. Therefore, the physiological and functional significance of the conserved Ski7_C_ is unclear. Here, we report strong genetic evidence suggesting differential roles for Ski7_N_ and Ski7_C_ in normal and specific mRNA turnover pathways by creating/isolating mutations in both Ski7_N_ and Ski7_C_ conserved motifs using indicator yeast strains. We concluded that Ski7_C_ participates in mRNA surveillance as a regulatory module competitively with the Hbs1/Dom34 complex. Our results provide insights into the molecular regulatory mechanisms underlying mRNA surveillance.

Translational guanosine triphosphatases (GTPases) are a protein superfamily involved in various steps of protein synthesis. The elongation factor (EF)1α GTPase subfamily paralogues, such as EF1α, eRF3, and Hbs1 function in translation elongation, termination, and mRNA surveillance, respectively. These factors, in their guanosine triphosphate (GTP)-bound conformations, bind with the tRNA or tRNA-mimicking proteins eRF1 and Pelota (Dom34 in yeast), respectively, to form structurally similar complexes. These complexes enter the ribosomal A site to participate in genetic decoding or ribosomal rescue steps in a GTP hydrolysis-dependent manner. It was recently revealed that the archaeal homologue of EF1α, aEF1α, binds 2 protein tRNA mimics, aRF1 and aPelota, in addition to tRNAs, suggesting a common GTPase-dependent mechanism underlying these processes^1^,^2^,^3^,^4^ (Supplementary Fig. S1).

The EF1α-like paralogue Ski7 was first identified as a member of the super killer (ski) antiviral system that blocks expression of non-poly(A) mRNA in the budding yeast *Saccharomyces cerevisiae*^5^,^6^,^7^. Ski7 is involved in an alternative yeast pathway of non-stop decay (NSD) for aberrant mRNA surveillance^8^,^9^ and cooperates with the Ski complex and the cytoplasmic exosome that facilitates 3′-to-5′ mRNA decay^10^. Double-mutant strains such as *xrn1*Δ *ski7*Δ, *dcp1 ski7*Δ, and *dcp2 ski7*Δ, in which 5′-to-3′ exoribonuclease (Xrn1)^7^ or mRNA 5′-decapping enzyme subunits (Dcp1/Dcp2)^11^ responsible for common 5′-to-3′ mRNA decay are defective, exhibit synthetic lethality. Because at least the 3′-to-5′ or the 5′-to-3′ mRNA decay pathway is required for yeast survival, these evidences strongly suggest that Ski7 participates in the general 3′-to-5′ decay pathway as well as in the mRNA surveillance system for aberrant mRNA decay. However, the molecular basis for each distinctive mechanism is unclear.

Ski7 consists of an N-terminal (Ski7_N_) and a C-terminal (Ski7_C_) region. Ski7_C_ shares overall sequence similarity with the translational GTPase EF1α family proteins. Phylogenetic analysis revealed that Hbs1^12^, which is involved in the no-go decay mRNA surveillance system (NGD) as well as NSD, is the closest paralogue of Ski7^13^. One characteristic feature of Ski7_C_ is the substitution of a conserved histidine residue with serine in the G3 motif that is crucial for GTP hydrolysis^14^. Ski7 interacts with the Ski complex and the exosome via different regions of Ski7_N_. Analysis of a whole-Ski7_C_-deletion mutant, which solely expresses Ski7_N_, revealed that the Ski7_N_ domain is required and sufficient for 3′-to-5′ mRNA decay^8^,^10^. The C-terminal domain was speculated to play specific roles in the degradation of nonstop mRNAs^8^ by analysis of the whole-Ski7_C_-deletion mutant and a whole-Ski7_N_-deletion mutant solely expressing Ski7_C_. Those studies, using N-/C-terminally truncated proteins, gave important clues to the domains’ function. However, single-amino-acid mutants, which would reveal the specific functional domains of Ski7, haven’t been studied yet. Hence, the molecular dissection of the functional domains of the full-length Ski7 protein at the level of amino acid residues has been awaited.

Recently, high-resolution X-ray crystal structures of Ski7_C_ complexed with either intact GTP or GDP-Pi were solved^15^. The structural conformation including the major nucleotide-binding residues of both forms is quite similar to that of GTP-bound forms of other GTPase paralogues, except for some residues involved in interactions with the γ-phosphate of GTP. Furthermore, purified Ski7_C_ did not exhibit GTP hydrolytic activity, even in the presence of 80S ribosome with/without eRF1/Dom34, suggesting the existence of a yet-unknown Ski7 cofactor that facilitates GTP hydrolysis. Although the structural study of Ski7_C_ provided a clear insight into the highly conserved as well as exceptional feature of Ski7 as a member of translational GTPases, it failed to provide any clue for its functional relationships with the Ski7_N_ domain in the full-length Ski7 protein. Therefore, the functional meaning of the conserved C-terminal GTPase-like domain attached to the essential N terminus remains unsolved.

In this study, we successfully identified a series of NSD loss-of-function mutations in both Ski7_N_ and Ski7_C_ via systematic mutagenesis and genetic screening. Structural mapping and additional *in vivo* analyses suggested that Ski7_C_ plays a specific role in mRNA surveillance as a regulatory module through its G-domain function.

## Results

### Identification of a functional amino acid residue in Ski7_N_ by alanine scanning

**Figure 1:**
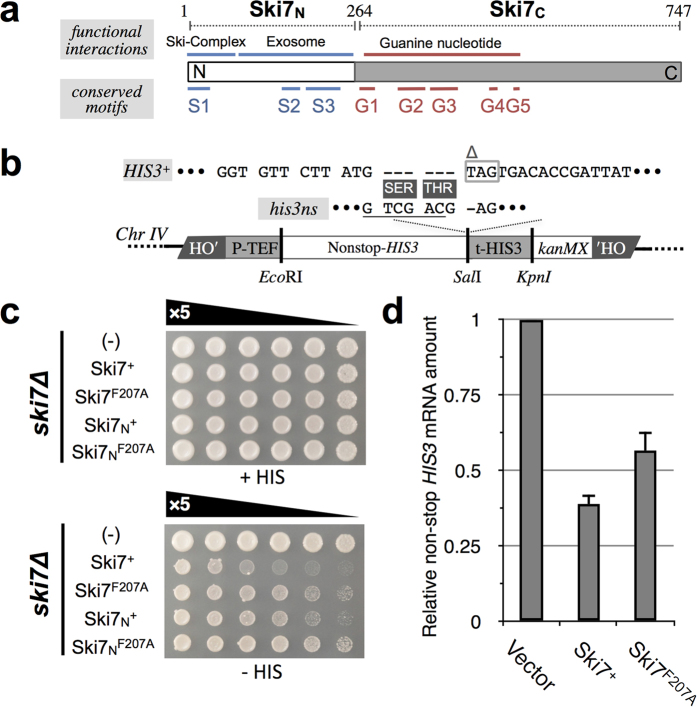
(**a**) Schematic representation of the N-terminal and C-terminal domains and known conserved motifs of Ski7 protein from *S. cerevisiae.* S1–S3: conserved motifs found in the N-terminal domain of Ski7 (Ski7_N_)^15^. G1–G5: GTPase consensus motifs commonly found among translational GTPases in the C-terminal domain of Ski7 (Ski7_C_)^43^. Regions for binding with Ski2-Ski3-Ski8 complex (Ski complex); the exosome complex and guanine nucleotides are also indicated. (**b**) Schematic representation of the non-stop *HIS3* (*his3ns*) reporter unit integrated in the genome of the assay strains. For details, see Methods. (**c**) Spot growth assay identified F207A in the N-terminal domain of Ski7 as a loss-of-function mutation. Defect of NSD caused by F207 mutation was confirmed in the full-length as well as in the Ski7_N_ form. YWH-1*ski7*Δ transformants (the relevant genotype is denoted as “*ski7Δ*” on the left) of p414CYC (TRP1 marker)-based Ski7 expression vectors (“Ski7^+^”: p414CYC-SKI7(WT), “Ski7^F207A^”: p414CYC-SKI7-F207A, “Ski7_N_^+^” p414CYC-SKI7_N_(WT), “Ski7_N_^F207A^”: 414CYC-SKI7_N_-F207A), including an empty vector control, were spotted on SC-TRP (+HIS) and SC-TRP-HIS plates and cultured for 3 days at 30 °C as described in the Methods. Results for the entire set of N-terminal mutations in motifs S2 and S3 are shown in Supplementary Fig. S3. (**d**) Relative mRNA amount of the non-stop *HIS3* reporter in the WT (Ski7^+^) and F207A mutant of full-length Ski7 transformants shown in (C) as compared to the vector control ( = 1) determined by qPCR. Data represent the mean ± SD (n = 3).

To study the NSD-facilitating activity of Ski7, each of the amino acid residues within the N-terminal conserved S2 and S3 motifs of Ski7 (Ski7_N_) were systematically substituted with alanine (Fig. 1a). These motifs were selected on the basis of multiple sequence alignment analysis of Ski7 homologues from various fungal species and are part of the putative exosome-interacting region^16^. First, an assay strain was constructed by integrating a non-stop *HIS3* reporter gene construct, which expresses aberrant non-stop mRNA substrate suitable for NSD, into the genomes of a *his3**∆** ski7**∆***strain (BY4727 background)^17^ (Fig. 1b). The assay strain (hereafter designated as YWH-1*ski7*Δ) was transformed with vectors expressing full-length, wild-type (WT) Ski7, Ski7_C_ and Ski7_N_, respectively. The full-length Ski7 transformant exhibited a severe growth defect on yeast synthetic medium lacking histidine (−HIS) due to rapid degradation of the aberrant non-stop *HIS3* mRNA via the NSD pathway. In contrast, the blank vector transformant grew normally since the mRNA was stabilized in the absence of Ski7-dependent NSD activities. The Ski7_N_ fragment was functional, though seemingly a bit weakly, while the Ski7_C_ fragment was not effective as compared to the blank vector control (Supplementary Fig. S2a)^8^. Cellular expression of the Ski7 variants was confirmed by western blotting using anti-Ski7 antibody (Supplementary Fig. S2b).

Next, a series of Ski7 expression vectors harbouring single alanine substitutions was introduced into YWH-1*ski7*Δ and the activities of Ski7_N_ mutations versus the WT were assessed via histidine auxotrophic growth of the transformants. Among the 20 Ski7_N_ mutants, only the F207A mutant exhibited significant growth enhancement on assay plates as compared to *SKI7*^+^ (Fig. 1c, Supplementary Fig. S3), suggesting loss of NSD function. The mutational effect was also observed with Ski7 lacking the C-terminal region (Fig. 1c: Ski7_N_^+^, Ski7_N_^F207A^). Stabilization of the non-stop *HIS3* mRNA in the F207A transformant was consistently confirmed by quantitative polymerase chain reaction (qPCR) (Fig. 1d). F207A is the first single amino acid loss-of-function mutation of Ski7 reported to date, though unexpectedly from the conservation patterns of the motifs.

### Genetic isolation of NSD loss-of-function mutations in Ski7_C_

**Figure 2:**
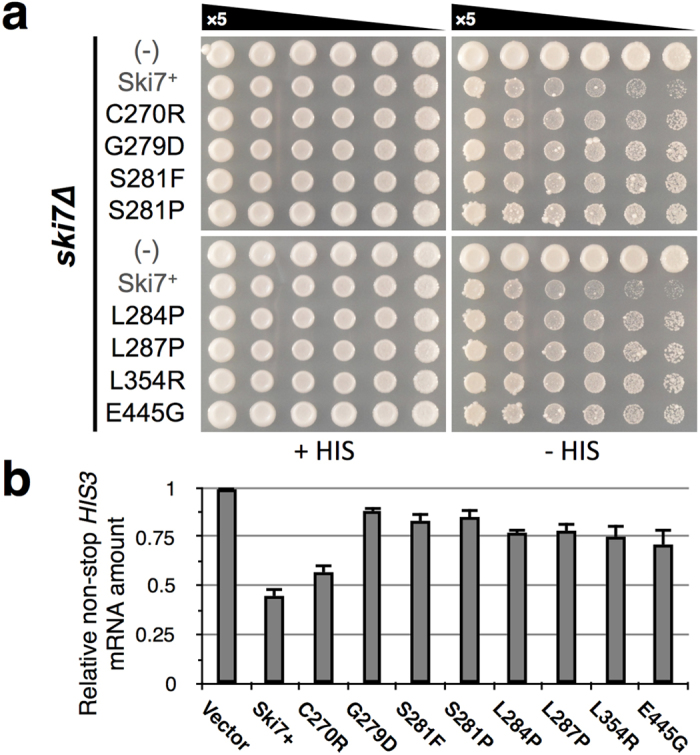
(**a**) Spot growth assay of the loss-of-function mutations in Ski7_C_ generated by random mutagenesis. YWH-1*ski7*Δ transformants (denoted as “*ski7Δ*” on the left) of WT and mutant full-length Ski7 expression vectors (p414CYC-SKI7(WT)-based), including an empty vector control, were spotted on SC-TRP (+HIS) and SC-TRP-HIS (−HIS) plates and cultured at 30 °C for 4 days. (**b**) Relative amount of non-stop *HIS3* mRNA in the WT (Ski7+) and mutant full-length Ski7 transformants shown in (A) as compared to the vector control ( = 1) determined by qPCR. Data represent the mean ± SD (n = 3).

**Figure 3:**
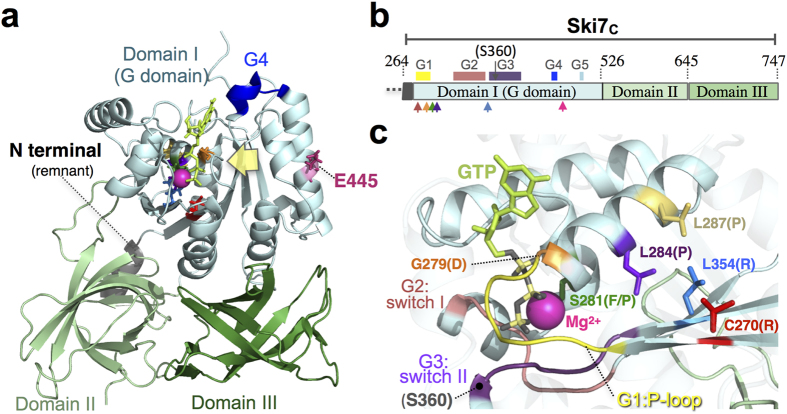
Three-dimensional, structural mapping of the Ski7 mutations in the Ski7_C_ region. (**a**) Overall structure of Ski7_C_ bound to GTP (pdb id: 4ZKE)^15^. The three structural domains of Ski7 are depicted using cartoon models in pale cyan (Domain I or G Domain), pale green (Domain II) and green (Domain III). The locations of the mutated residues are shown in the X-ray crystal structure of Ski7 using a stick model with colours distinct from that of the backbone. The distant location of E445, apart from the GTP-binding region, is indicated. The residual part of the N-terminal region adjacent to the Ski7_C_ region is shown in grey. GTP is shown using a stick model in lime green. The catalytic magnesium ion is shown as a pink sphere. The G4 motif is shown in blue. (**b**) Schematic representation of the domain arrangement of Ski7_C_ subdomains. The positions of the 5 conserved sequence motifs (G1–G5) in Domain I are highlighted with coloured boxes on top. Arrows in respective colours at the bottom indicate the mutated positions. (**c**) Close-up view of the GTP-binding regions of Domain I of Ski7 from the direction indicated by the yellow arrow in (A). The positions of the mutations as well as other residues in the text are labelled. The phosphotriester moiety of GTP is shown in grey (oxygen) and yellow (phosphate). Crucial G Domain elements are also indicated; P-loop (G1 motif) in yellow, Switch I (G2 motif) in salmon pink, and Switch II (G3 motif) in purple.

Next, we attempted to isolate mutations among the single amino acid residues of Ski7_C_ aiming to elucidate their functional significance in NSD. Using modified, error-prone PCR (see Methods, Supplementary Fig. S4), random *SKI7* mutations were accumulated on the expression vector (p416TEF-SKI7(WT), *URA3* marker) and introduced into the assay strain. Loss-of-function mutations of Ski7 were screened for enhanced growth of YWH-1*ski7*Δ on SC-URA-HIS plates. Approximately 250 candidate colonies were isolated from independent experiments and *SKI7*-encoding sequences of the recovered plasmids were determined. Next, 153 candidate plasmids were introduced into YWH-1*ski7*Δ for reproducibility testing. Most of the recovered plasmids were shown to be pseudo-positive, probably due to a high reversion frequency of the assay strain background, in which the non-Ski7 NSD pathway remained active. In total, 10 positive survivor clones contained multiple mutational sites (approximately 7 on average with some overlap) across the complete *SKI7* coding region. Each of the single mutations detected in the C-terminal region were then introduced separately into the original p416TEF-SKI7(WT) by site-directed mutagenesis and tested for activity in YWH-1*ski7*Δ. Consequently, 8 single mutations (C270R, G279D, S281F, S281P, L284P, L287P, L354R, and E445G, in 7 different amino acid loci in total, Supplementary Fig. S5) in the full-length construct of *SKI7* reproducibly resulted in a significant loss-of-function phenotype (Fig. 2a). These mutant proteins were expressed in yeast cells at a level comparable to that of the wild-type Ski7 (Supplementary Fig. S6). Consistently, stabilization of the non-stop *HIS3* mRNA of the transformants was confirmed by qPCR (Fig. 2b). All of these mutations were located within or in the close vicinity of GTP-binding motifs (Supplementary Fig. S5, Fig. 3b).

Next, the 7 mutational sites were mapped on the X-ray crystal structure of the GTP-bound form of Ski7 (pdb id: 4ZKE)^15^. The results showed that all mutations were located in the C-terminal structural domain I of the Ski7 protein (Fig. 3a,b). Intriguingly, except for E445, all residues were clustered near the GTP-binding site and were structurally related to each other (Fig. 3c). G279 and S281 formed possible contact sites for the phosphotriester moiety of GTP and the magnesium ion on the opposite site, respectively, while the side chains of C270, L284, L287, and L354 were clustered with face-to-face contacts right behind the guanine nucleotide binding site. These results suggested that these mutations may have caused conformational, kinetic, and/or putative catalytic disorders related to guanine nucleotide binding. On the other hand, E445 was located distant from the guanine nucleotide binding site of domain I on the side chain directly outwards of this domain, in the outer α-helix. The corresponding residues in other canonical translational GTPases on the ribosomes most likely form crucial contacts with the ribosomal components^18^,^19^.

To examine the functional relationship between the isolated mutations, double and triple mutants of Ski7_C_ were constructed and subjected to NSD spot assays and western blotting analysis (Supplementary Fig. S7). The combinatorial mutations hardly enhanced the degree of loss of function or affected the expression level of the mutant protein, indicating that each single mutation is sufficient and redundant for complete knockout of a certain specific function in Ski7_C_. In contrast, Ski7 harbouring double mutations in both Ski7_N_ and Ski7_C_ exhibited more severe loss-of-function phenotypes than either single mutant (Supplementary Fig. S8). These observations provided genetic evidence for distinct molecular functions of Ski7_N_ and Ski7_C_ in NSD.

### Dominant-negative and Hbs1/Dom34-competitive action of Ski7_C_ mutations in NSD

**Figure 4:**
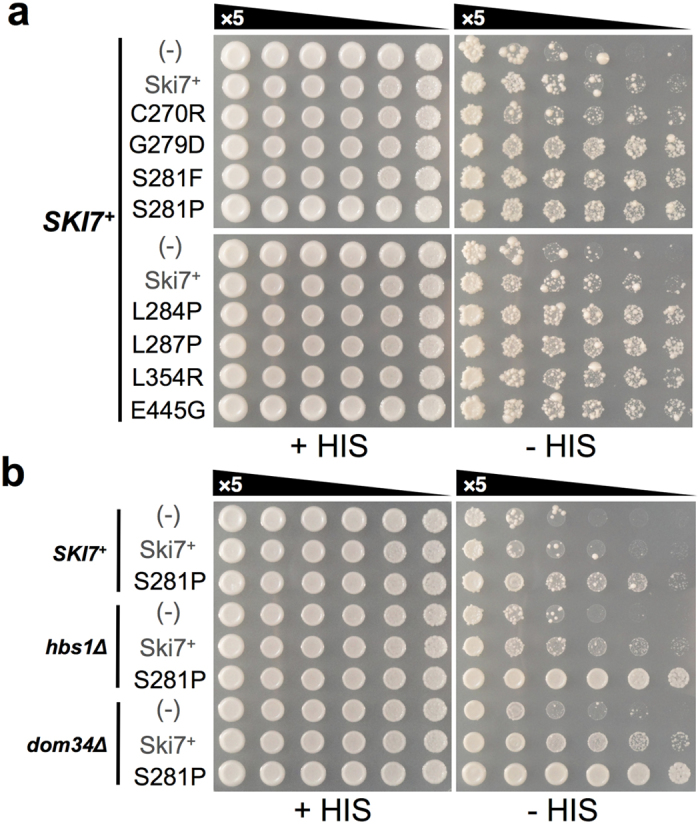
(**a**) Dominant-negative effects of Ski7 loss-of-function mutations examined by spot growth assay. YWH-1*SKI7*^+^ transformants (denoted as “*SKI7*^+^” on the left) of empty vector, WT Ski7, and mutant full-length Ski7 expression vectors (p414GPD-SKI7(WT) based) were spotted onto spitted onto SC-TRP (+HIS), SC-HIS-TRP (-HIS) plates and grown at 30 °C for 4 days as described in the Methods. (**b**) The Hbs1/Dom34 effect on WT Ski7 and the S281P Ski7 mutant was examined in the dominant-negative assay strain lacking either Dom34 (YWH-1*dom34*Δ) or Hbs1 (YWH-1*hbs1*Δ) by spot growth assay for 3 days. Strain genotypes are denoted as *SKI7*^+^ (YWH-1*SKI7*^+^ strain), *hbs1Δ* (YWH-1*hbs1*Δ strain), and *dom34Δ* (YWH-1*dom34Δ* strain) on the left.

Because NSD is a translation-coupled quality control system that involves various translational GTPases other than Ski7, Ski7_C_ mutants are likely to cause functional defects in the ribosome. To evaluate this, the dominant-negative effect on NSD was examined in the presence of WT Ski7 on the genome. The Ski7_C_ mutants were sub-cloned into the higher expression vector p414GPD^20^ and introduced into the assay strain YWH-1*SKI7*^+^, which is isogenic to YWH-1*ski7Δ*^20^ (Table S1). Even in the presence of WT Ski7 on the chromosome, 7 out of 8 mutations (G279D, S281F, S281P, L284P, L287P, L354R, and E445G) resulted in increased growth on assay plates lacking histidine, indicating that they are dominant-negative (Fig. 4a). The C270R mutant inhibited NSD more weakly than the other mutants, which was consistent with its NSD activity level within the cell (Fig. 2a,b).

Genetic, biochemical, and structural analyses using G-domain mutations^22^,^23^,^24^,^25^,^26^ and non-hydrolysable GTP analogs^27^,^28^,^29^,^30^ of translational GTPases have shown that conformational changes in translational GTPase assisted by guanine binding regulate their ribosomal binding mode. Thus, G-domain-specific defects often cause prolonged stalling on the ribosome, resulting in dominant-negative inhibition of competitive processes, such as nucleotide hydrolysis or exchange. Accordingly, we speculated that G-domain mutants of Ski7 would cause certain ribosomal functional defects and remain on the ribosome, resulting in the inhibition of competitive binding of the WT Ski7 and probably, of the closest-related GTPase Hbs1 as well. Hbs1 binds to the ribosome in a ternary complex with GTP and Dom34 to contribute to NGD and NSD^31^,^32^,^33^. Thus, to evaluate our hypothesis, the dominant-negative effect of the Ski7_C_ mutation (S281P) was further tested in isogenic assay strains lacking either *HBS1* or *DOM34* (YWH-1*hbs1Δ* and YWH-1*dom34*Δ). The effect of *hbs1Δ* or *dom34Δ*, *per se*, in this assay strain was cryptic (Fig. 4B (−) spots in *SKI7*^+^ vs. *hbs1Δ* or *dom34Δ*). However, intriguingly, when WT Ski7 was over-expressed in the *hbs1Δ* or *dom34Δ* reporter strain, a small but significant dominant-negative effect on NSD was observed (Fig. 4b, Ski7^+^ spots in the *hbs1Δ* and *dom34Δ* genetic background). This result suggested that Ski7 functions in NSD in a competitive and concerted way with Hbs1/Dom34, even under normal conditions, i.e. these factors share a functional site on the ribosome with other translational GTPases. Furthermore, the dominant-negative effect of S281P was strikingly enhanced in the absence of Hbs1 or Dom34 (Fig. 4b). The other Ski7_C_ mutants consistently exhibited similar effects in *dom34Δ* genetic background (Supplementary Fig. S9). Together, these results supported our hypothesis.

Furthermore, the dominant-negative effects of the Ski7_C_ mutants as well as that of the WT were nullified in their respective Ski7_C_-only forms (Supplementary Fig. S10a), while the cellular expression levels of the truncated Ski7_C_ mutants were comparable to that of the wild type (Supplementary Fig. S10b). These results suggested that molecular functions of Ski7_C_, at least those operated by the Ski7_C_ amino acid residues revealed in this study, are effective absolutely in the presence of Ski7_N_, i.e. in the full-length form.

### Synthetic lethality of Ski7_N_ and Ski7_C_ mutations in combination with xrn1Δ

**Figure 5:**
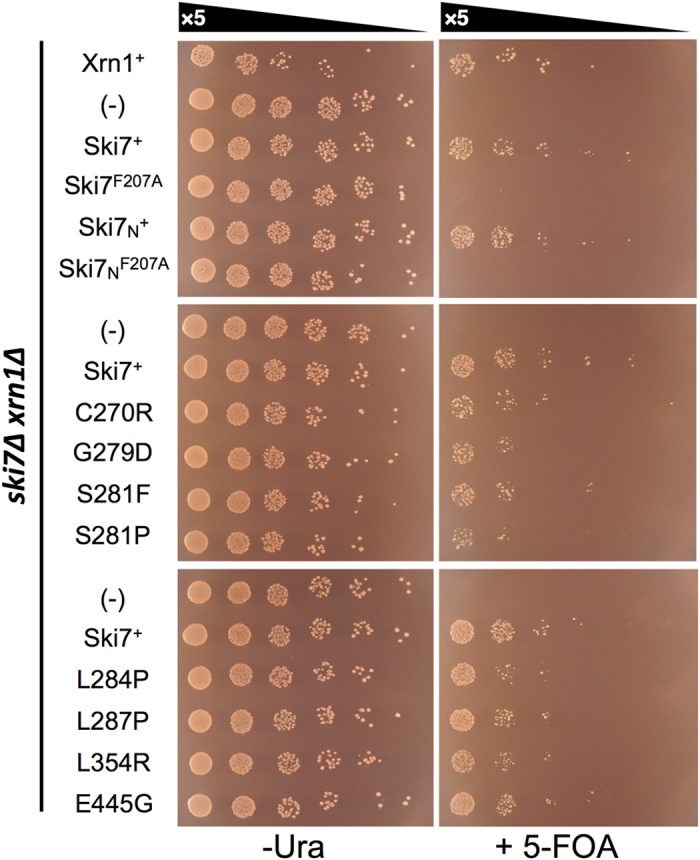
Spot growth assays for Ski7_N_ and Ski7_C_ mutations in combination with *xrn1Δ*. Double-knockout cells (*ski7∆xrn1∆*) carrying expression vectors for Xrn1 (p416GPD-Xrn1, *URA3* marker) and full-length Ski7 mutants (p413CYC-SKI7(WT)-based, *HIS3* marker) were 5-fold serially diluted and spotted on SC-HIS-LEU-URA (−URA) and SC-HIS-LEU with 0.6 g/L 5-FOA (+5-FOA) plates. The cells were cultured at 30 °C for 3 days. Xrn1^+^ and (−) denote Xrn1-expressing vector (p413GPD) and empty vector (p413CYC), respectively.

Finally, we investigated the function of Ski7 mutations in normal mRNA turnover. The double-knockout strain *xrn1∆ski7∆* is known to exhibit synthetic growth defects, suggesting that Ski7 plays a crucial role in normal mRNA turnover as well as in NSD. Thus, to assess whether Ski7 mutants rescued these growth defects, the *xrn1∆ski7∆* double-knockout strain harbouring an Xrn1 expression vector (hereafter designated as YKE-XRN1) was transformed with a series of vectors expressing Ski7 variants (p413CYC-based, *HIS3* marker) or Xrn1 as a control (p413GPD-based, *HIS3* marker). The growth of the transformants was assessed on URA3 counter-selective medium containing 5-fluoroorotic acid (5-FOA), in which only transformant cells with sufficient Ski7 activity in the absence of Xrn1 can grow normally. Under these conditions, transformants harbouring Xrn1, Ski7, or Ski7_N_ plasmids grew normally (top panels, Fig. 5). The F207A mutation in either full-length Ski7 or Ski7_N_ construct did not allow for rescue of the growth defect (top panels, Fig. 5), whereas 8 single mutations in domain I of Ski7_C_ showed comparable rescue effects (middle and bottom panels, Fig. 5). These results indicated that the loss-of-function mutants remained active in normal mRNA turnover. Taken together with the results of the NSD assays (Fig. 4a), the physiological function of Ski7_C_ appears to be more specific to NSD pathway and distinct from that in normal mRNA turnover, as suggested previously^8^,^10^, and the G-domain of the Ski7_C_ plays a crucial role in this function.

## Discussion

In this study, we adopted different genetic strategies to isolate loss-of-function mutations to identify crucial amino acid residues of Ski7_N_ and Ski7_C_ in NSD (Fig. 1a). Alanine substitution mutagenesis in Ski7_N_ identified the F207A mutation in motif S3 of Ski7^16^ constituting the putative exosome-binding region (Fig. 1c,d). This mutation seemed to confer general loss of mRNA degradation function (Fig. 5). On the other hand, random mutagenesis by error-prone PCR identified 7 independent residues in domain I of Ski7_C_ (Fig. 2a,b). Importantly, except for E445, these residues were located in the core guanine-binding region. Moreover, the side chains of these 6 residues were clustered in the X-ray structure of Ski7_C_. This suggests that these residues constitute a switch region for conformational change of Ski7 in response to guanine nucleotide binding. Additionally, 7 out of the 8 Ski7_C_ mutations (G279D, S281F/P, L284P, L287P, L354R, and E445G) clearly exhibited dominant-negative NSD inhibition in the presence of WT Ski7 (Fig. 4a). Moreover, the dominant-negative effect on NSD, in part by the Ski7_C_ mutations as well as by the WT, was greatly enhanced in the absence of Hbs1 or Dom34, which form a putative ribosomal rescue complex in mRNA surveillance pathways. Importantly, the Ski7_N_-truncated forms of the Ski7_C_ mutants lost their dominant-negative activity even in the *dom34*Δ background strain (Supplementary Fig. S4). These finding imply a competitive mechanism of action of Ski7 and the Hbs1/Dom34 complex on the ribosome in NSD, which could be regulated by certain conformational changes corresponding to the G-domain status of Ski7_C_ in the presence of the adjacent Ski7_N_ domain. Furthermore, the Ski7_C_ mutations, unlike the F207A in Ski7_N_, did not exhibit loss of function in synthetic lethality with *xrn1∆* (Fig. 5), suggesting that Ski7_C_ is predominantly necessary for NSD but is not strictly required for normal mRNA turnover.

**Figure 6:**
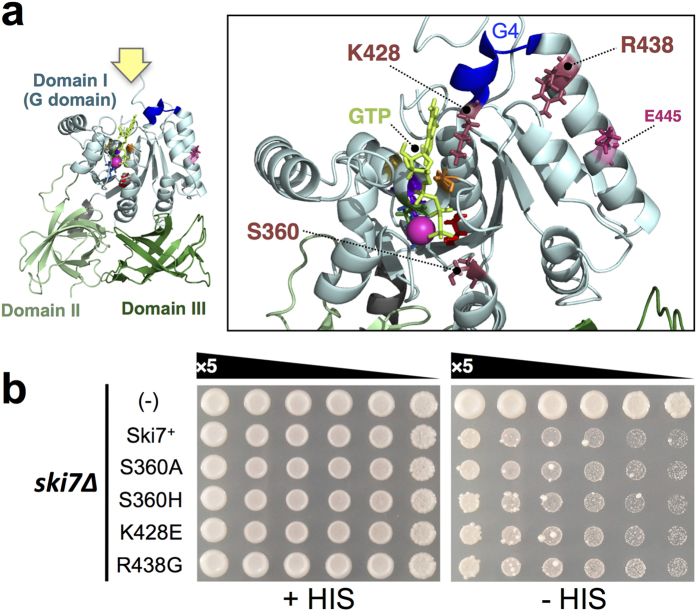
Spot growth assay of mutation of crucial amino acid residues in the G domain. (**a**) The overall structure of Ski7_C_ is shown on the left. Close-up view from above (yellow arrow) is shown on the right. Positions of S360, K428, and K438 are shown in the Ski7_C_ crystal structure (in raspberry red). Other information is as in Fig. 3. (**b**) Spot growth assay of WT as well as S360A, S360H, K428E, and K438G mutants of full-length Ski7. YWH-1*ski7Δ* transformants of the p414CYC-based expression vectors for full-length WT (Ski7^+^) and S360A, S360H, K428E, and K438G mutants, including an empty vector control, were spotted on SC-TRP (+HIS: left panel) and SC-TRP-HIS (−HIS: right panel) plates and cultured at 30 °C for 4 days.

To confirm the importance of the G domain of Ski7_C_, 4 single mutations were introduced in 3 amino acid residues that are known to be crucial for GTP hydrolysis/binding in other translational GTPases^34^,^35^,^36^, i.e. S360A, S360H, K428E, and R438G (Fig. 6a,b). However, those 4 mutations exhibited minimal, if any, loss of NSD function.

Therefore, it can be speculated that the 8 Ski7_C_ mutations isolated in this study caused loss of function at one certain step of the Ski7 mechanism, at which the adjacent Ski7_N_ domain is required. However, it is plausible that the prominent feature of these mutations enabled the mutant strains to be predominantly selected, despite the high background noise of the assay systems (Supplementary Fig. S4).

Here, it is worth asking what triggered the change in the ribosome-binding mode of Ski7. In a previous study^15^, highly purified Ski7_C_ did not exhibit GTP-hydrolytic activity with the 80S ribosome in the presence or absence of tRNA-mimicking protein adaptors such as eRF1 and Dom34. As mentioned previously^14^, a substitution of the conserved histidine by serine (S360 in Ski7) in the G3 motif in other EF1α paralogues totally abolished GTP-hydrolytic activity. Else, Ski7 may require a genuine specific adaptor(s) for recognition of mRNAs targeted for NSD by triggering the activation of GTP hydrolysis in the ribosome. Unfortunately, no mutation was isolated in the adaptor-binding regions of Ski7_C_ domains II and III (Supplementary Fig. S1) in the Ski7 loss-of-function mutation screening in this study. Further genetic screening of mutants may demonstrate such possibilities, based on this study; nevertheless, other scenarios cannot be excluded.

The properties of the mutations may provide some clues. Firstly, E445 was found on the α-helix harbouring intermolecular contact sites, and this helix was connected to the G4 motif (Figs 3a and 6a), which contains crucial residues for guanine base recognition. Secondly, 6 out of the 8 mutations (C270R, G279D, S281F/P, L284P, and L287P) in this study were clustered around the G1 motif (also termed “P-loop”), which stably bound to the β-phosphate moiety of GTP and GDP before and after hydrolysis in canonical GTPases^15^. L354 made close contact with L284 and L287 in the local structure. Thus, all mutations in this study can be speculated to affect, directly or indirectly, guanine binding rather than putative catalytic activity for GTP hydrolysis.

Domain I is the first structural constituent of translational GTPases to connect to the extra N-terminal domains in EF1α paralogues such as Hbs1 and eRF3. The N-terminal region adjacent to the G domain has been reported to affect the conformation and function of the C-terminal regions^37^. The N-terminal extra region of eRF3 exhibited a unique intra-molecular interaction with the C-terminal region (eRF3c) in crystal structures^37^. Guanine binding hardly affected the crystal structures, suggesting potential regulatory functions of the N-terminal portion of translational GTPases for the conformation of the C-terminal part and partner molecule binding. Intermolecular interaction through the N-terminal region was shown to affect the overall activities of eRF3^38^. However, in the case of Ski7, dysfunction caused by Ski7_C_ mutation was observed only in the presence of Ski7_N_ (Supplementary Fig. S10), suggesting that the functional conformation was assisted by the N-terminal region. Furthermore, it has been reported that the bacterial translational GTPase RF3, in its GDP-binding form, binds the ribosome without any adaptors and releases RF1 and RF2 by affecting the ribosomal conformation. The subsequent ribosomal conformational change accelerates the GDP-to-GTP exchange by RF3 via intermolecular interactions, to recycle itself from the ribosome by GTP hydrolysis^39^,^40^. Therefore, it is tempting to assume that inter- or intramolecular interactions with Ski7c on the ribosome would mechanically switch the binding mode of Ski7 for guanine nucleotides without hydrolysis in the sequential steps of NSD. Further *in vivo* and *in vitro* analyses are required to test this hypothesis.

In summary, we successfully identified amino acid residues crucial to Ski7 function in its N- and C-terminal regions. Ski7_C_ mutations suggested their specific involvement in NSD and differential roles in mRNA surveillance and normal mRNA turnover in the cell. Further detailed biochemical and structural analyses are necessary; however, those mutations could be an important clue for revealing unidentified molecular aspects of Ski7, an eccentric EF1α homologue.

## Methods

### Yeast media

Synthetic complete (SC) medium was prepared with appropriate dropout mix (ForMedium) lacking amino acids essential for plasmid transformants. For plates, 2% agar was added.

### Plasmid constructions

The SKI7 fragment including *Spe*I and *Sal*I restriction sites at the 5′ and 3′ ends, respectively, was amplified by PCR using primers SKI7-N/SKI7-C (for full-length), SKI7_C_-N/SKI7-C (for SKI7_C_), and SKI7-N/SKI7_N_-C (for SKI7_N_) using BY4727 genomic DNA as a template. The DNA fragment digested with *Spe*I and *Sal*I was ligated into the *Spe*I-*Sal*I sites of the p414CYC (for low expression; TRP1 marker)/p416TEF (for moderate expression; URA3 marker)/p414GPD (for high expression; TRP1 marker) or the p416CYC (for low expression; URA3 marker) plasmid vectors^20^.

### Construction of non-stop HIS3 reporter strain

The *HIS3* gene coding region (non-stop *HIS3*) and its own transcription termination region (t-HIS3) containing known polyadenylation sites were amplified with PCR primers HIS3-N/His3ns-C and HIS3t-ns-5/HIS3t-3 using *HIS*+ yeast genomic DNA as a template. The 2 DNA fragments were digested with restriction enzymes at both ends as indicated (Table S3), and inserted downstream of the TEF promoter site of the HO locus integration vector pHO-poly-kanMX4-HO^41^ derivative pHO-TEF-kanMX-HO as depicted in Fig. 1b. The *HIS3-*coding region on the vector contains an additional *Sal*I restriction site at the end, thus adding serine and threonine residues, and does not contain an in-frame stop codon before the 3′ end of poly(A) due to a well-known single-base deletion mutation in the stop codon TAG^8^. This HIS3ns reporter construct was integrated at the *HO* gene locus on the yeast chromosome IV as described previously^41^.

### mRNA quantitation by qPCR

To confirm the effects of Ski7 mutations on the decay of non-stop mRNA, total RNA of each mutant was prepared from yeast cells that were cultured to an OD_600_ of 1.0. RNA was isolated using RNAiso Plus (Takara Bio Inc.) with shaking, with glass beads on a FastPrep 24 (MP Biomedicals Inc.). qPCR was performed with the Power SYBR Green PCR Master Mix (Applied Biosystems) via reverse transcription using PrimeScript RT Master Mix (Takara Bio Inc.) and cDNA amplification using the following primers: RT-HIS3-F and RT-HIS3-R for the non-stop *HIS3* reporter, and RT-ACT1-F and RT-ACT1-R for *ACT1*, encoding cytoskeletal actin, used as an internal control. qPCR data were acquired on a 7900HT Fast Real-Time PCR System (Applied Biosystems).

### Isolation of loss-of function mutations of Ski7 using error-prone PCR

A DNA fragment containing full-length *SKI7* as well as the downstream half of *URA3* was amplified by error-prone PCR with primers RM-URA3-F/RM-PTEF-R. The complementary part of the plasmid containing the upstream half of *URA3* was amplified with Phusion High-Fidelity DNA Polymerase (Thermo Fisher Scientific K.K.) with primers RM-URA3-R/RM-PTEF-F using p416TEF-SKI7(WT) as a template. The 2 DNA fragments contained an overlapping region of 150–200 bp. The DNA fragments were mixed and co-introduced into the assay strain YWH-1*ski7*Δ to regenerate p416TEF-SKI7 by homologous recombination. Cells were cultured on a SC-URA-HIS plate for up to 7 days at 30 °C as shown in Supplementary Figure S4.

### Spot growth assay

Transformed cells were cultured in SC-TRP liquid medium at 30 °C overnight. The next day, the liquid samples were diluted 1:10 with the same medium and grown until the OD_600_ reached 1.0. Then, the samples were 5-fold serially diluted and spotted onto SC-TRP (for non-selective control) and SC-HIS-TRP (for selection) plates. To test synthetic lethality, transformed cells grown on SC-HIS-LEU-URA plate were suspended in sterile water and the suspension was diluted to an OD_600_ of 0.25. Subsequently, 5-fold serial dilutions were prepared and spotted onto plates containing non-selective SC-HIS-LEU-URA medium and selective SC-HIS-LEU medium with 0.6 g/L 5-FOA. The plates were incubated at 30 °C for several days as indicated in the legends.

### Site-directed mutagenesis

Single mutations of Ski7 were introduced by site-directed mutagenesis of the WT or mutant Ski7 plasmid vectors using appropriately designed primers as described previously^42^.

### Western blotting

Whole-cell extracts were prepared from yeast cells cultured to an OD_600_ of 1.0, resolved by sodium dodecyl sulphate-polyacrylamide gel electrophoresis (10% polyacrylamide), and transferred to polyvinylidene fluoride membranes (Merck Millipore). The transferred proteins were stained with anti-Ski7_NC_ polyclonal antibody (mixture of polyclonal antibody raised against Ski7_N_ peptide (103–119) and polyclonal antibody against Ski7_C_ peptide (479–495)) and anti-PGK antibody (Invitrogen, Thermo Fisher Scientific K.K.). Antibodies were visualized using enhanced chemiluminescence western blotting detection reagents (GE Healthcare Bio-Sciences K.K.). Images were captured with a LAS-3000 mini imaging system (Fujifilm).

## Additional Information

**How to cite this article**: Horikawa, W. *et al*. Mutations in the G-domain of Ski7 cause specific dysfunction in non-stop decay. *Sci. Rep.* **6**, 29295; doi: 10.1038/srep29295 (2016).

## Supplementary Material

Supplementary Information
