# Supplementary material for: Mutations in the G-domain of Ski7 cause specific dysfunction in non-stop decay
Source: Sci Rep. 2016 Jul 6;6:29295. doi: 10.1038/srep29295 (PMC4933942; doi:10.1038/srep29295)
Supplement: Supplementary Information [file srep29295-s1.pdf]

Supplementary Information

Mutations in the G-domain of Ski7 cause specific dysfunction in non-stop decay

Wataru Horikawa[1], Kei Endo[1], Miki Wada[1, 2] & Koichi Ito[1]*

[1]Department of Computational Biology and Medical Sciences, Graduate School of Frontier Sciences, The University of Tokyo, Kashiwa-city, Chiba 277-8562, Japan

[2]Technical office, The Institute of Medical Science, The University of Tokyo, Minato-ku, Tokyo 108-8639, Japan

* To whom correspondence should be addressed. Tel: +81 4 7136 3600; Fax: +81 4 7136 3601; Email: itokoichi@k.u-tokyo.ac.jp

Supplementary Figures S1–S10

Supplementary Tables S1–S3

Supplementary References

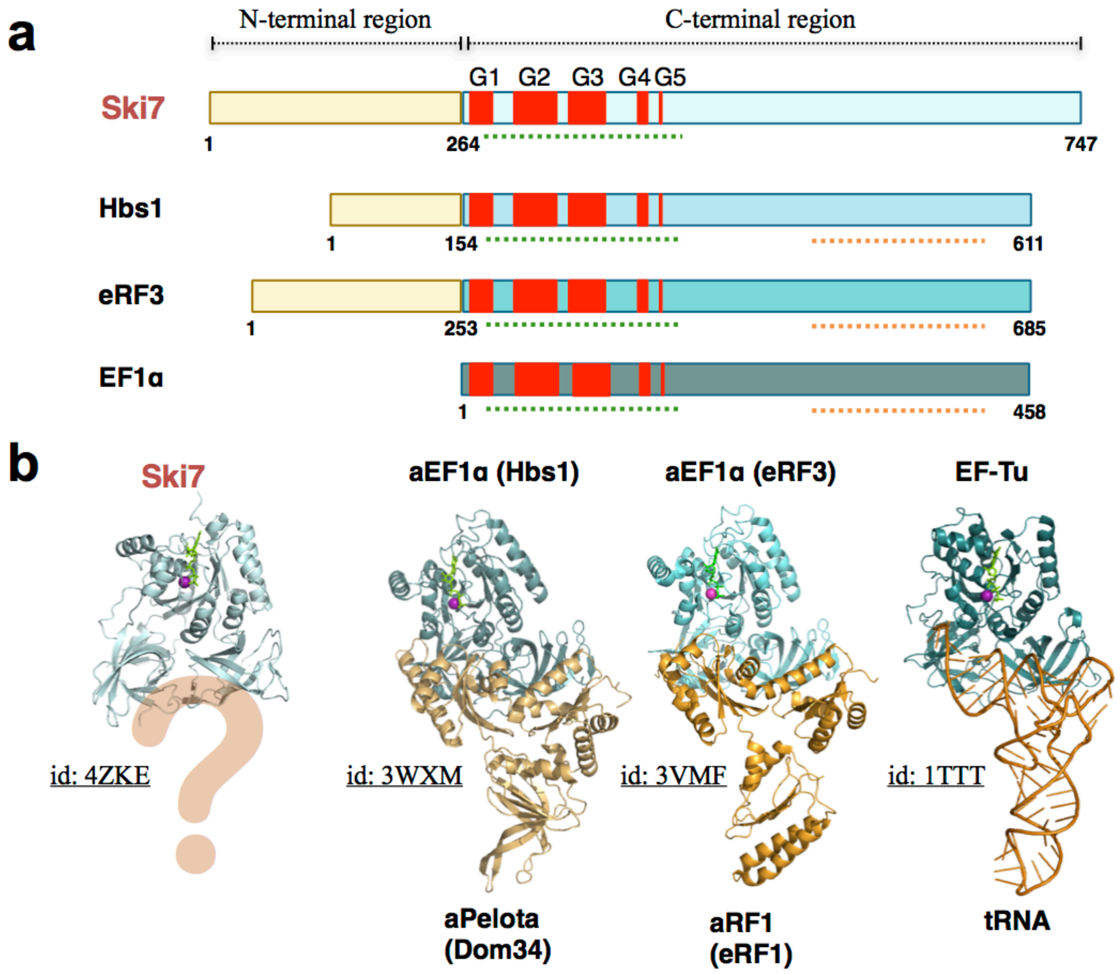

Figure S1. Ski7 and EF1α paralogues in Eukaryotes and Archaea. (a) Schematic domain organization of Ski7 and other EF1α paralogues in *Saccharomyces cerevisiae*. Relevant domains of EF1α paralogues in *Saccharomyces cerevisiae* are shown; Ski7 (NP_014719), Hbs1 (NP_013010.3), eRF3 (NP_010457.3) and EF1α (NP_015405.1). G domains are underlined with green dotted lines and the consensus motifs (G1–G5) are shown as red boxes. The approximate partner binding regions (Pelota/Dom34 for Hbs1, eRF1 for eRF3, and tRNAs for EF1α) are underlined with orange dotted lines. **(b)** Crystal structures of Ski7 and tertiary complexes of other EF1α paralogues with tRNA/tRNA mimicking proteins and GTP (analogue). Ski7 (pdb id: 4ZKE) are shown on the left. The binding partner molecules corresponding to tRNA or tRNA mimicking proteins are not known for Ski7 (thus shown as orange "?"). aPelota/aEF1α/GTP complex (pdb id: 3WXM), aRF1/aEF1α/GTP complex (pdb id: 3VMF) and bacterial tRNA/EF-Tu/GDPNP complex (pdb id: 1TTT) on behalf of orthologous Dom34/Hbs1/GTP complex, eRF1/eRF3/GTP complex and tRNA/EF1α/GTP, respectively, as complete structures for those complexes are not available. EF1α paralogues are shown in cyanish colours and binding partners are in orangey colours.

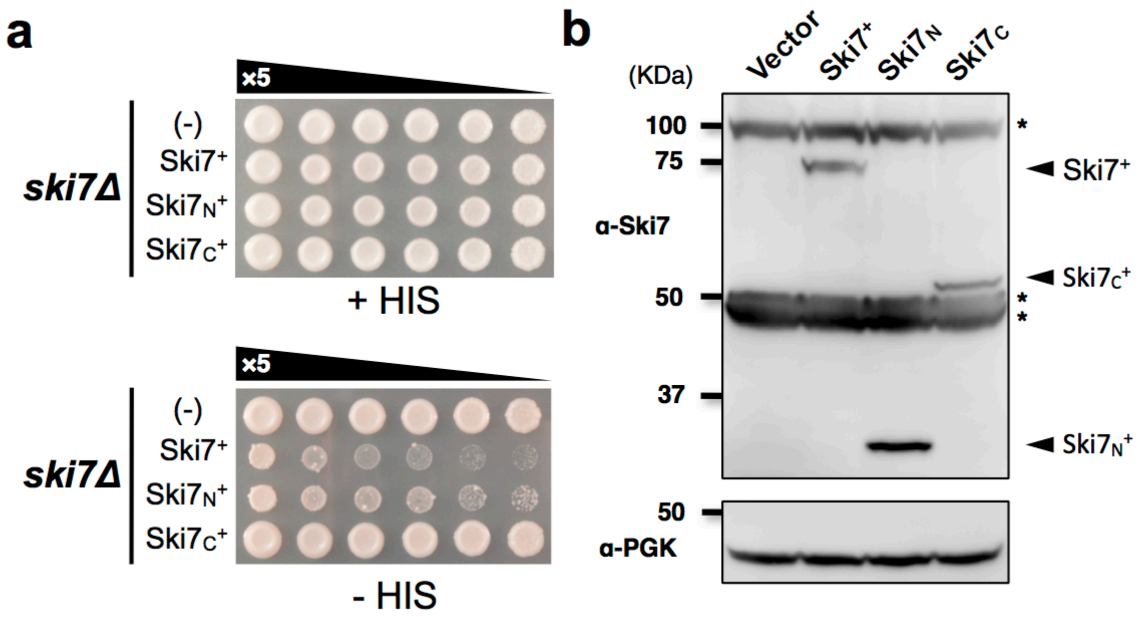

Figure S2. (a) Spot growth assay of Ski7 variants in NSD. YWH-1*ski7*Δ transformants of the p414CYC-based expression vectors for full-length Ski7 ($Ski7^+$), the N-terminal domain only ($Ski7_N^+$) and the C-terminal domain only ($Ski7_C^+$) were spotted on SC-URA (+HIS: upper panel) and SC-TRP-HIS (-HIS: lower panel) plates and cultured at 30°C for 4 days. The activity of each domain for NSD was essentially reproduced as reported previously[8]. **(b)** Detection of the full-length, $Ski7_N^+$, and $Ski7_C^+$ proteins in the yeast cells as in (a) by western blot analysis. Expression levels of Ski7 variants were detected as described in the Methods. PGK protein was detected by reprobing the membranes with anti-PGK antibodies as a loading control. Asterisks indicate nonspecific bands.

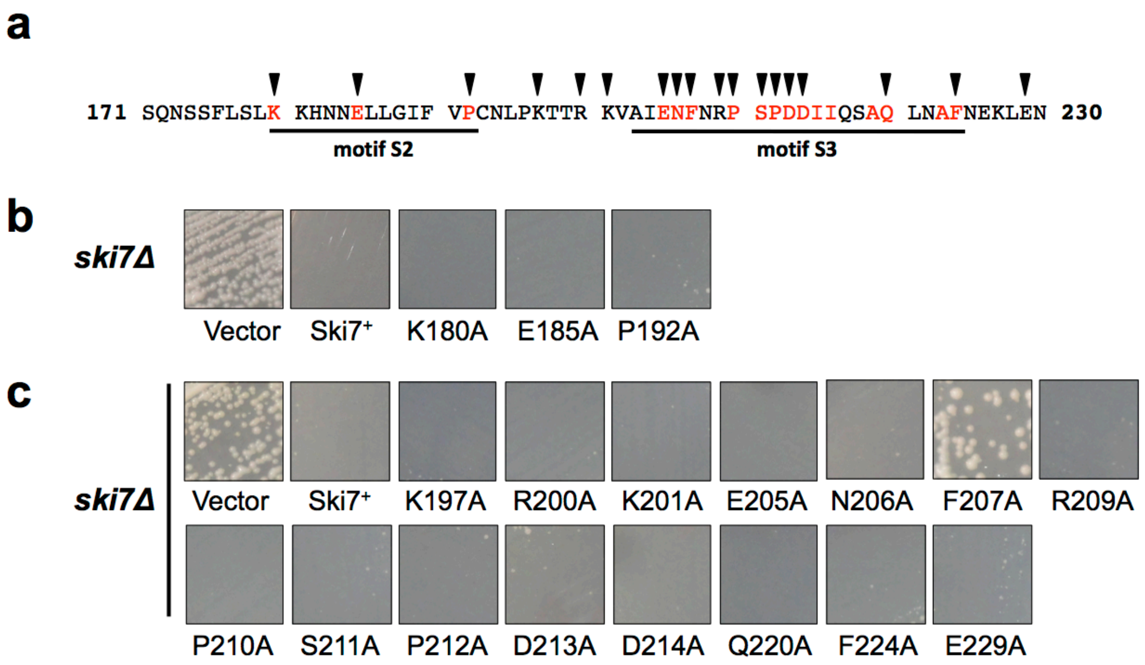

Figure S3. NSD activity test for the alanine substitution mutations in conserved motifs of N-terminal region of Ski7. (a) Amino acid residues 171–230 of Ski7 containing the conserved motifs S2 and S3 (underlined) proposed by Marshall *et al.*[16]. Well-conserved residues within motifs S2 and S3 are shown in red. The positions of the alanine-substituted residues are indicated with arrows. **(b)** NSD colony formation assay of the Ski7 mutations in motif S2. YWH-1*ski7*Δ transformants of the p416CYC based expression vectors for wild-type and mutant full-length Ski7, including an empty vector control, were streaked on a SC-URA-HIS plate and cultured at 30°C for 7 days. **(c)** NSD colony formation assay of the Ski7 mutations in motif S3. YWH-1*ski7*Δ transformants of the p416CYC-based expression vectors for WT and mutant full-length Ski7, including an empty vector control, were streaked on the SC-URA-HIS plate and cultured at 30°C for 7 days.

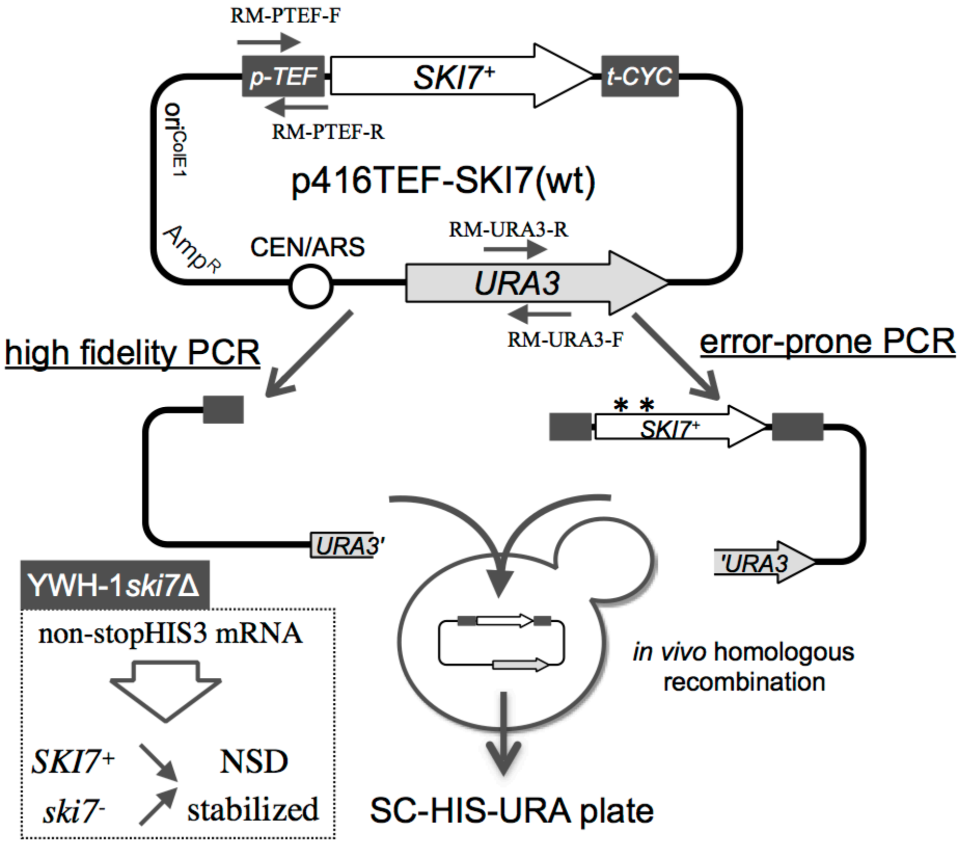

Figure S4. Modified random mutagenesis by error-prone PCR

In our preliminary trials, it was shown that screening for loss-of-function mutations of Ski7 in the plasmid vector, in which the error-prone PCR mutagenized *Ski7* fragment was directly ligated into the expression vector p416TEF to reconstitute p416TEF-SKI7(WT), resulted in pseudo-positive candidates. This can be principally explained by 2 reasons; (i) High reversion frequency of the NSD assay strain, and (ii) non-negligible rate of malformed plasmid vector generated by vector self-ligation reaction. The assay strain (YWH-1*ski7*Δ) is designed to optimally detect Ski7 activity by selecting the promoter unit (TEF) for the non-stop HIS3 reporter. Even so, however, NSD activity by non-Ski7 pathways remaining in the cell as well as histidine auxotrophic growth are frequently affected directly/indirectly by numerous spontaneous mutations in the genetic background. Thus, to overcome high background noise caused by (ii), we used modified random mutagenesis with error-prone PCR as depicted in this figure. A DNA fragment containing the full-length *Ski7* gene as well as the downstream half of *URA3* was amplified by error-prone PCR with primers RM-URA3-F/RM-PTEF-R (right), while a complementary DNA fragment containing the upstream half of *URA3* was amplified with Phusion High-Fidelity DNA Polymerase (Thermo Fisher Scientific K.K., Japan) with DNA primers RM-URA3-R/RM-PTEF-F using p416TEF-SKI7(WT) as a template (left). Both DNA

fragments contained an overlap of 150–200 bp. The two DNA fragments were mixed and co-introduced into the assay strain YWH-1*ski7*Δ to regenerate p416TEF-SKI7 by homologous recombination. Cells were cultured on a SC-HIS-URA plate at 30°C for up to 7 days. Most of the positive candidates contained multiple mutations. Though some mutations seemed to be effective in combination with others, we tested each single-mutation by site-directed mutagenesis and selected only singly effective mutations in this study.

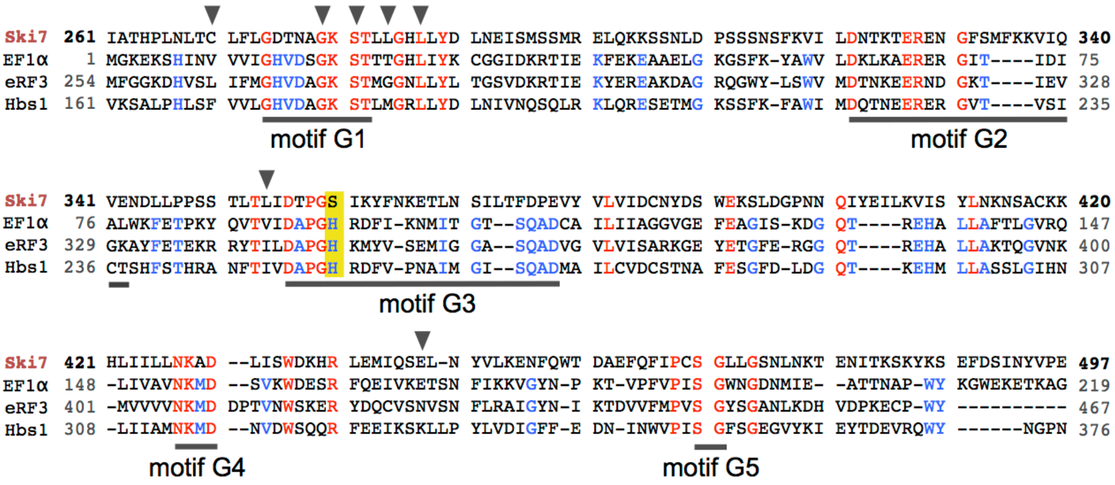

Figure S5. Multiple sequence alignment of domain I of the EF1α paralogues in *S. cerevisiae*. The amino acid sequences of EF1α paralogues in budding yeast, Ski7 (*SKI7*), EF1α (*TEF1*), eRF3 (*SUP35*), and Hbs1 (*HBS1*) are aligned by homology. Positions of the 5 conserved motifs (G1–G5) are underlined, while the locations of the 7 loss-of function mutations isolated in this study are indicated by arrows. Conserved residues among all homologous proteins and homologous proteins except for Ski7, are shown in red and blue, respectively. S360 and its corresponding residues in other paralogues are highlighted in yellow.

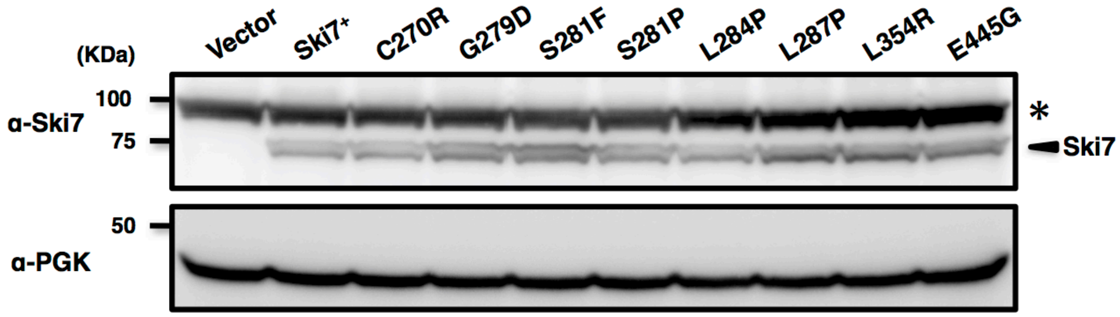

Figure S6. Detection of the full-length wild-type and Ski7$_C$ mutants in the yeast cells by western blotting. Expression levels of Ski7 variants were detected as described in the Methods. PGK protein was detected by reprobing the membranes with anti-PGK antibodies as a loading control. Asterisks indicate nonspecific bands.

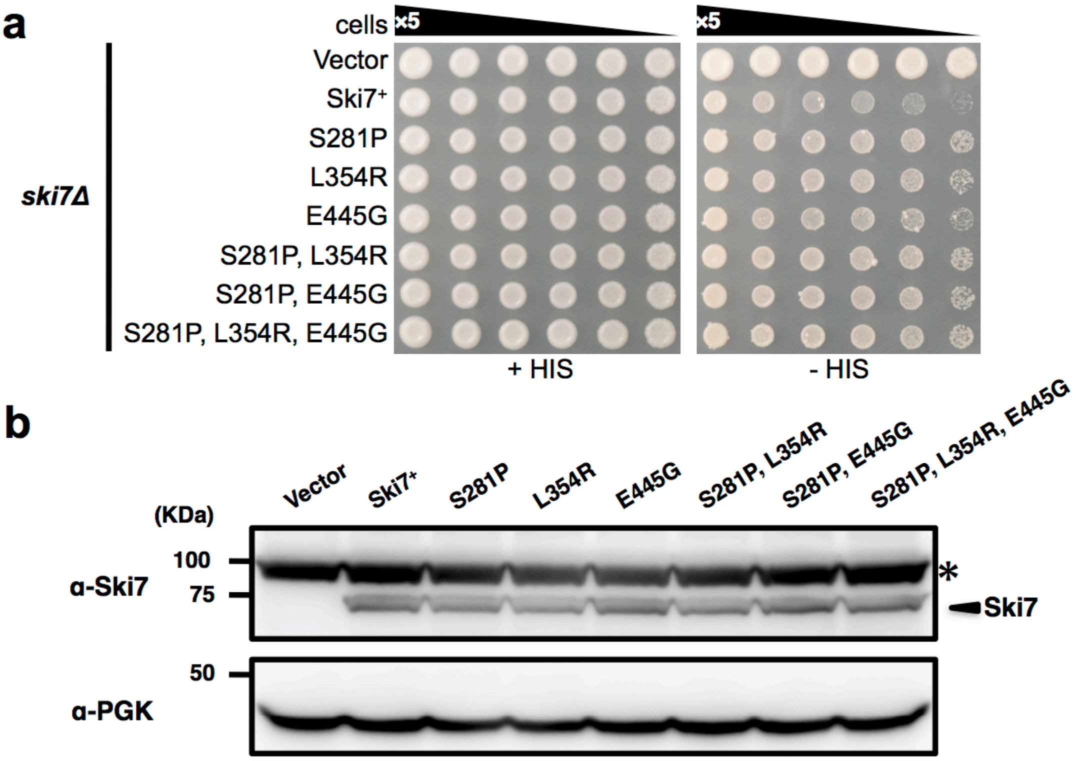

Figure S7. (a) Spot growth assay of multiply mutated Ski7$_C$ in NSD. YWH-1*ski7*Δ transformants (denoted as "*ski7Δ*" on the left) of WT, singly (S281P, L354R, E445G), doubly (S281P/L354R, S281P/E445G) and triply (S281P/L354R/E445G) mutated Ski7 expression vectors (p414CYC-SKI7(WT) based), including a an empty vector control, were spotted on SC-TRP (+HIS) and SC-TRP-HIS (-HIS) plates and cultured at 30°C for 4 days. **(b) Cellular expression of the full-length wild type and Ski7$_C$ mutants in (a) detected by western blotting analysis**. Expression levels of Ski7 variants were detected as described in the Methods. PGK protein was detected by reprobing the membranes with anti-PGK antibodies as a loading control. Asterisks indicate nonspecific bands.

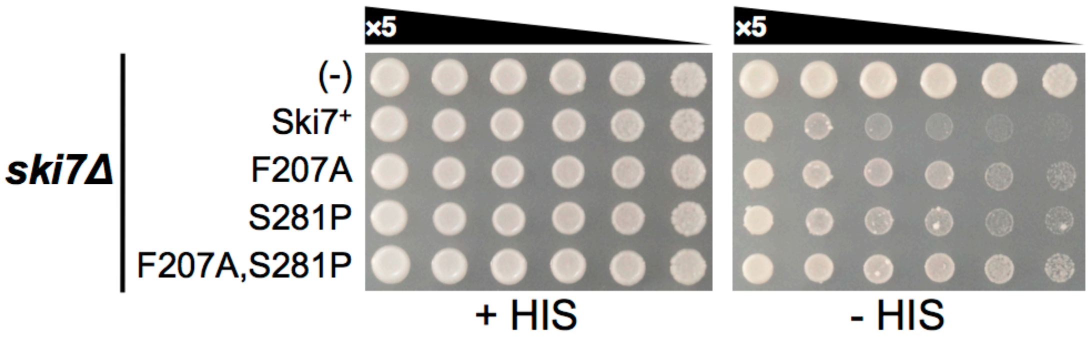

Figure S8. Spot growth assay of combinational mutants of F207A, S281A in NSD. YWH-1*ski7*Δ transformants of the p414CYC-based expression vectors for full-length wild type (Ski7^{+}) and singly and doubly mutated Ski7 by F207A (in Ski7$_{N}$ region) and S281P (in Ski7$_{C}$ region), including an empty vector control, were spotted on SC-TRP (+HIS: left panel) and SC-TRP-HIS (-HIS: right panel) plates and cultured at 30°C for 3 days.

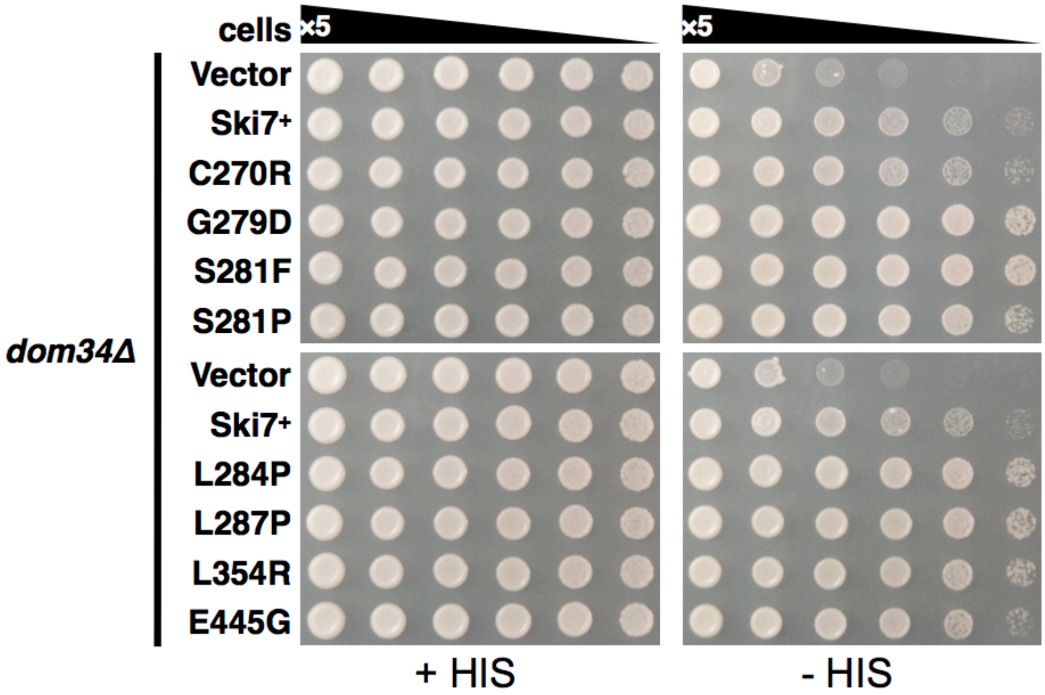

Figure S9. Spot growth assay of Ski7$_C$ mutants in the YWH-1*dom34*Δ strain. YWH-1*dom34*Δ transformants (denoted as " *dom34*Δ" on the left) of empty vector, WT Ski7, and mutant full-length Ski7 expression vectors (p414GPD-SKI7(WT) based) were spotted onto SC-TRP-HIS (+HIS), SC-TRP (-HIS) plates and grown at 30°C for 2 days as described in the Methods.

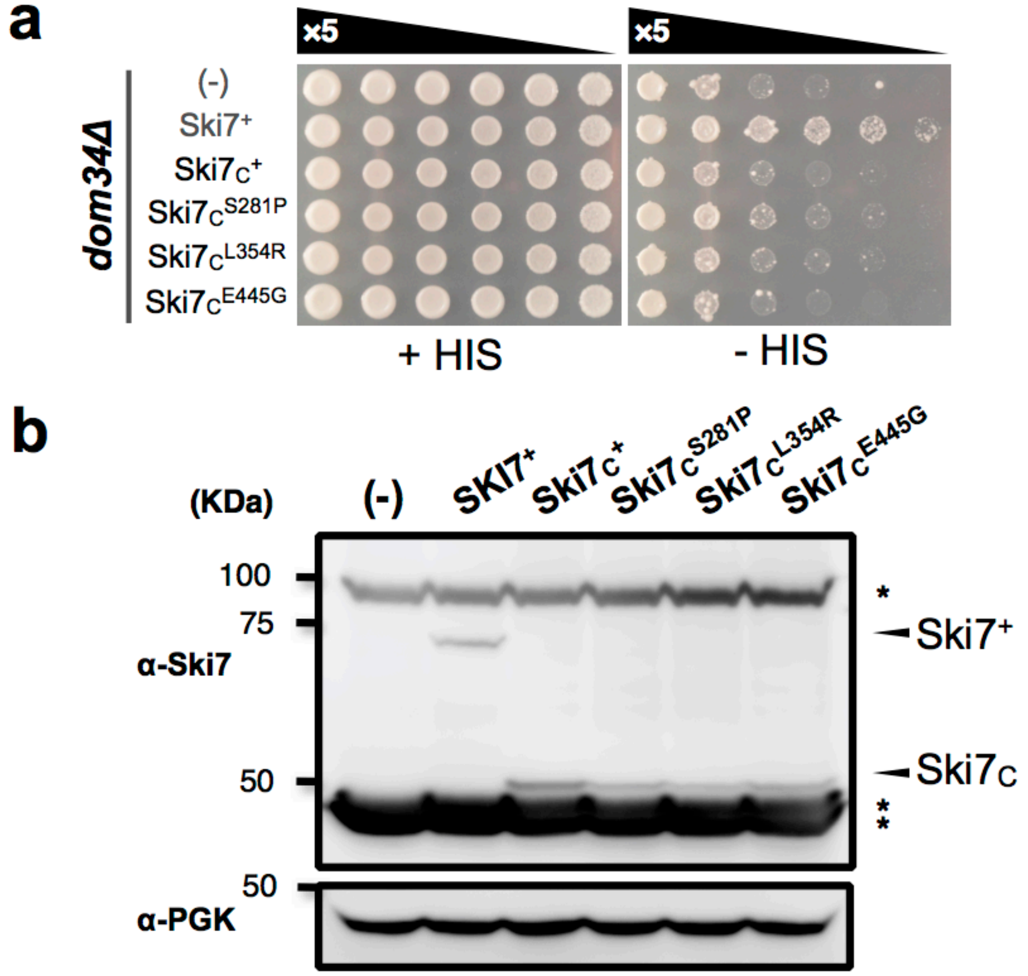

Figure S10. (a) Spot growth assay of Ski7$_C$ mutations in the N-terminal truncated form. YWH-1*dom34*Δ transformants of the p416GPD based Ski7$_C$ expression vectors for WT, S281P, L354R, E445G mutations (p416GPD-SKI7$_C$(WT)-based), including a an empty vector control, were spotted on SC-URA (+HIS: left panel) and SC-URA-HIS (-HIS: right panel) plates and cultured at 30°C for 3 days. **(b) Detection of the full-length and Ski7$_C$ proteins from the expression vectors (as in (a)) in the yeast strain YWH-1*dom34*Δ by western blot analysis.** Expression levels of Ski7 variants were detected as described in the Methods. PGK protein was detected by re-probing the membranes with anti-PGK antibodies as a loading control.

Table S1: Yeast strains used in this study.

Name of Strain	Genotype	Reference
**NSD reporter assay strains**		
BY4727	*MATα his3Δ200 leu2Δ0 lys2Δ0 met15Δ0 trp1Δ63 ura3Δ0* (S288C: parental strain)	17
BY4727*ski7Δ*	*MATα his3Δ200 leu2Δ0 lys2Δ0 met15Δ0 trp1Δ63 ura3Δ0 ski7::hphMX*	This study
YWH-1*SKI7*$^{+}$	*MATalpha his3Δ200 leu2Δ0 lys2Δ0 met15Δ0 trp1Δ63 ura3Δ0 HO::TEFp-HIS3ns-HIS3t-kanMX*	21
YWH-1*ski7*Δ	*MATalpha his3Δ200 leu2Δ0 lys2Δ0 met15Δ0 trp1Δ63 ura3Δ0 ski7::hphMX HO::TEFp-HIS3ns-HIS3t-kanMX*	This study
YWH-1*hbs1*Δ	*MATalpha his3Δ200 leu2Δ0 lys2Δ0 met15Δ0 trp1Δ63 ura3Δ0 hibs1::hphMX HO::TEFp-HIS3ns-HIS3t-kanMX*	This study
YWH-1*dom34*Δ	*MATalpha his3Δ200 leu2Δ0 lys2Δ0 met15Δ0 trp1Δ63 ura3Δ0 dom34::hphMX HO::TEFp-HIS3ns-HIS3t-kanMX*	This study
**Ski7 synthetic lethality assay strain**		
BY4742	*MATα his3Δ1 leu2Δ0 lys2Δ0 ura3Δ0* (S288C: parental strain)	17
YKE1-XRN1 [*1]	*MATα his3Δ1 leu2Δ0 lys2Δ0 ura3Δ0 ski7::hphMX xrn1::LEU2* p416GPD-XRN1^{+}	This study

*1 While the phenotype of *ski7Δxrn1Δ* double-knockout strain reported[7] is very sick or conditionally lethal, it is strictly synthetically lethal in BY4742. The slight difference in phenotypic behaviours could be caused by minor differences in the backgrounds of laboratory strains[44,45].

Table S2: Plasmid vectors used in this study.

Name of Plasmid	Explanation*1	Reference
**Expression shuttle vectors**		
p413CYC	CYC promoter CEN6/ARSH4 HIS3 (Low expression)	20
p414CYC	CYC promoter CEN6/ARSH4 TRP1 (Low expression)	20
p416TEF	TEF promoter CEN6/ARSH4 URA3 (Medium expression)	20
p413GPD	GPD promoter CEN6/ARSH4 HIS3 (High expression)	20
p414GPD	GPD promoter CEN6/ARSH4 TRP1 (High expression)	20
p414CYC-SKI7(WT)*2	full-length wild-type (WT) Ski7	This study
p414CYC-SKI7-F207A	full-length WT Ski7 with F207 mutation	This study
p414CYC-SKI7$_N$(WT) *2	N terminal domain of WT Ski7 (denoted as Ski7$_N$)	This study
p414CYC-SKI7$_N$-F207A	Ski7$_N$ domain with F207 mutation	This study
p414CYC-SKI7$_C$(WT) *2	C terminal domain of WT Ski7 (denoted as Ski7$_C$)	This study
p413CYC-SKI7(WT) *2	full-length WT Ski7	This study
p416TEF-SKI7(WT) *2	full-length WT Ski7	This study
p416GPD-XRN1$^+$	WT Xrn1	This study
p413GPD-XRN1$^+$	WT Xrn1	This study
p414GPD-SKI7(WT) *2	full-length WT Ski7	This study
**Chromosome integration cassette plasmids**		
pHO-poly-KanMX4-HO	Yeast vectors for integration at the HO locus	41
pHO-TEF-kanMX-HO	pHO-poly-KanMX4-HO derivative with TEF promoter fragment with down-stream unique polylinker sites, *Bam*HI-*Sal*I-*Kpn*I.	This study

*1. Insert DNA fragments were amplified by PCR using the primers listed in Table S3, digested with restriction enzymes and inserted into restriction sites in the poly-linker site of the vectors as indicated in Table S3.

*2 Expression plasmids for Ski7 mutants created by site-directed mutagenesis are omitted in this list. They are essentially the same as the WT constructs except for mutations in Ski7 coding region.

Table S3: Primers used for the construction of plasmid vectors.

Name of Primers *1	Sequences (5' to 3')	Restriction site at 5' end (underlined) *2
	**For constructions of non-stop HIS3 reporter**	
HIS3-N	GGGAATTCCATATGACAGAGCAGAAAGCCCTAGTA	*Eco*RI
HIS3ns-C	ACGTCGACGCCATAAGAACACCTTTGGTGGAG	*Sal*I
HIS3t-ns-5	GCGTCGACGAGTGACACCGATTATTTAAAGCTGC	*Sal*I
HIS3t-3	GGGGTACCACTTGCCACCTATCACCACAACTA	*Kpn*I
	**For constructions of expression vectors for Ski7 variants**	
SKI7-N	GGACTAGTGGATCCATATGATGTCGTTATTAGAGCAATTAGCAAG	*Spe*I
SKI7-C	GGGTCGACTTACTGGCATGCAATTCTGC	*Sal*I
$\mathrm{SKI7_C}$-N	GGACTAGTGGATCCATATGCCTCTGAATTTGACATGTTTGTTCC	*Spe*I
$\mathrm{SKI7_N}$-C	GGGTCGACTTAATGGGTGGCAATGAATGAATGAATA	*Sal*I
XRN1-N	GGGGATCCATATGGGTATTCCAAAATTTTTCAGG	*Bam*HI
XRN1-C	GGGTCGACCTAAGTAGATTCGTCTTTTTTATTATCACGG	*Sal*I
	**For random mutagenesis of Ski7 plasmid**	
RM-PTEF-F	CTAGGGTGTCGTTAATTACCCGTAC	-
RM-PTEF-R	CTGAAACTTGAGAAATTGAAGACCG	-
RM-URA3-F	CGCAATGTCAACAGTACCCTTAGTA	-
RM-URA3-R	ACAGTCAAATTGCAGTACTCTGCGG	-
	**For mRNA quantitation by qPCR**	
RT-ACT1-F	TTGCCGAAAGAATGCAAAAG	-
RT-ACT1-R	GGAAGGTAGTCAAAGAAGCCAAGA	-
RT-HIS3-F	GACGACCATCACACCACTGAA	-
RT-HIS3-R	TCATCCAAAGGCGCAAATC	-

*1. “N” and “C” denote the N- and C-terminal positions of the open reading frame, respectively.

*2. Restriction sites for sub-cloning are indicated.

SUPPLEMENTARY REFERENCES

44. Schacherer, J. et al. Genome-wide analysis of nucleotide-level variation in commonly used *Saccharomyces cerevisiae* strains. *PLoS One.* **2**, e322 (2007).

45. Cubillos, F. A., Louis, E. J. & Liti, G. Generation of a large set of genetically tractable haploid and diploid Saccharomyces strains. *FEMS Yeast Res.* **9**, 1217-1225 (2009).
